# Conjugated Microporous Networks on the Basis of 2,3,5,6-Tetraarylated Diketopyrrolo[3,4-*c*]pyrrole^a^

**DOI:** 10.1002/marc.201100045

**Published:** 2011-04-15

**Authors:** Kai Zhang, Bernd Tieke, Filipe Vilela, Peter J Skabara

**Affiliations:** Department of Chemistry, University of CologneLuxemburger Str. 116, 50939 Cologne, Germany; WestCHEM, Department of Pure and Applied Chemistry, University of StrathclydeGlasgow G1 1XL, United Kingdom

## Abstract

π-Conjugated microporous networks have been prepared from the tetraarylated diketopyrrolo[3,4-*c*]pyrrole unit as a tetrafunctional building block. The reactions are carried out using microwave-assisted Yamamoto or Sonogashira cross-coupling. Red insoluble powders are obtained, showing intense fluorescence. The polymer networks exhibit a high gas storage capability, with BET surface areas up to about 500 m^2^ · g^−1^.

## Introduction

Microporous organic polymers (MOPs) such as polymers with intrinsic microporosity (PIMs),^1^ hyper-cross-linked polymers (HCPs),^2^ or covalent organic frameworks (COFs),^3^ have become of great interest in diverse application areas, such as gas separation,^4^ gas storage,2b,c, 4–5 or heterogeneous catalysis.^4^, ^6^ The application performance of these materials depends on the micropore structure and the physical surface area. The highest apparent Brunauer–Emmett–Teller (BET)^7^ surface areas achieved in amorphous MOPs are of the order of 2 000 m^2^ · g^−1^.^8^ Recently π-conjugated microporous materials have been gaining more and more attention.^9^ The first examples of microporous polymers (CMPs) with a fully π-conjugated system exhibiting high surface areas in the dry state were reported by Cooper et al. in 2007. The polymers consist of poly(aryleneethynylene) (PAE) with BET surface areas up to 834 m^2^ · g^−1^.9a Lately, Thomas et al. reported microporous conjugated networks based on spirobifluorene using Yamamoto polymerization, exhibiting surface areas larger than 1 000 m^2^ · g^−1^.^10^ Besides the usual application potentials for high surface area materials, for example, for gas storage or heterogeneous catalysis, conjugated polymer networks might be used in advanced fields such as optoelectronic applications.^11^ Recent activities are compiled in two review articles,^12^ and the most recent activities include microporous polymers based on spiro-bispropylene-dioxythiophene,^13^ and hydrocarbon polymers based on adamantane.^14^

**Figure:**
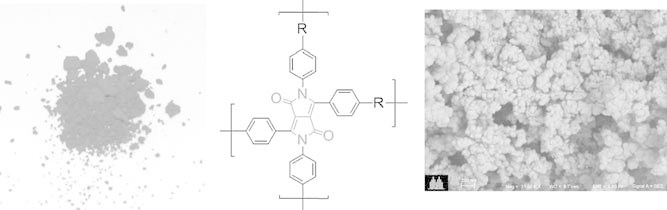


1,4-Diketopyrrolo[3,4-*c*]pyrrole, known as DPP, and some of its derivatives represent high-performance pigments used in paints, colored inks, and plastics.^15^ They show intense red colors and strong red photoluminescence,^15^, ^16^ which render them attractive as active materials in a variety of electronic devices, such as organic light-emitting diodes (OLEDs),^17^ organic field-effect transistors (OFETs),^18^ and organic solar cells.^19^ Because of the promising performance of DPP-based materials, there has been tremendous interest in investigating these materials in recent years.^20^

In this communication, we report on the first incorporation of DPP into conjugated porous networks with BET surface areas that range from 200 to 500 m^2^ · g^−1^ using microwave-assisted nickel-or palladium-catalyzed condensation reactions such as Yamamoto^21^ or Sonogashira–Hagihara^22^ cross-coupling. The polymers are characterized by high-resolution magic angle spinning (HR-MAS)-NMR, N_2_ gas sorption, fluorescence, and FT-IR spectroscopy. Supporting Information available. Synthetic routes of the starting compound *t-*BrDPP and the networks N1–N4,^27^ ^1^H and ^13^C NMR spectra of monomer *t-*BrDPP, HR-MAS-NMR spectra of the networks, SEM images of the networks, and pore size distributions are shown.

## Results and Discussion

**Scheme 1:**
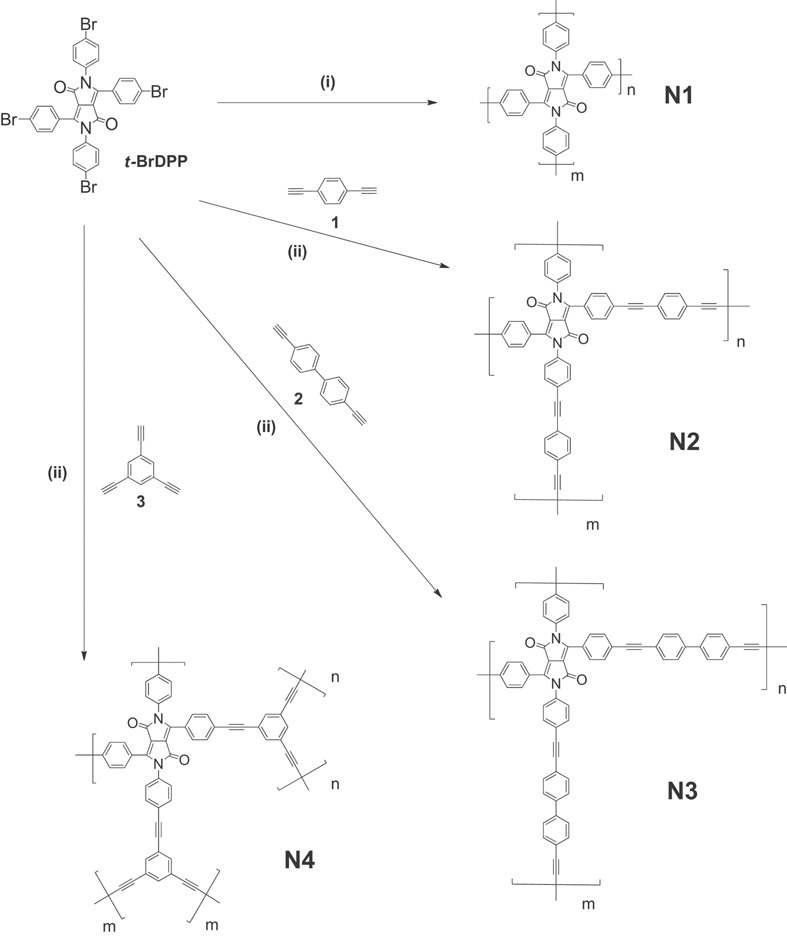
Synthesis and idealized structures of the polymer networks N1–4. Reagents and conditions: (i) Ni(COD)_2_, 2,2′-dipyridyl, cyclooctadiene, DMF, microwave, 100 °C,1 h. (ii) Pd(PPh_3_)_4_, NEt_3_, DMF, microwave, 100 °C, 1 h.

The monomers employed are listed in Table 1. The synthetic routes to the key materials are described in the Supporting Information. The Yamamoto-type self-condensation of 2,3,5,6-tetrakis(4-bromophenyl)pyrrolo[3,4-*c*]pyrrole-1,4(2*H*,5*H*)-dione (*t-*BrDPP) into polymer N1 was carried out using Ni(COD)_2_, 2,2′-dipyridyl, and cyclooctadiene. Polymers N2, N3, and N4 were synthesized upon palladium-catalyzed Sonogashira–Hagihara cross-coupling polycondensation of *t-*BrDPP with 1,4-diethynylbenzene (**1**), 4,4′-diethynyl-1,1′-biphenyl (**2**), and 1,3,5-triethynylbenzene (**3**), respectively (Scheme 1). The reactions were carried out under microwave-assisted conditions in a short period of 1 h. The polymers were precipitated as red powders, which were totally insoluble in all solvents investigated. Photographic images of the insoluble polymers are shown in Figure S7 of the Supporting information.

**Table 1:** Structural properties of networks N1–4.

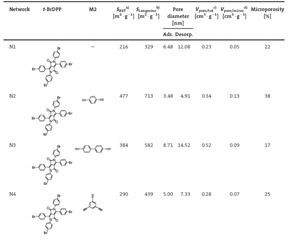

Surface areas calculated from the N_2_ adsorption isotherms using BET method;

Surface areas calculated from the N_2_ adsorption isotherms using Langmiur method;

Total pore volumes at *P/P*_0_ = 0.99;

Micropore volumes calculated using the *t*-plot method based on the Halsey equation.^26^

**Figure 1:**
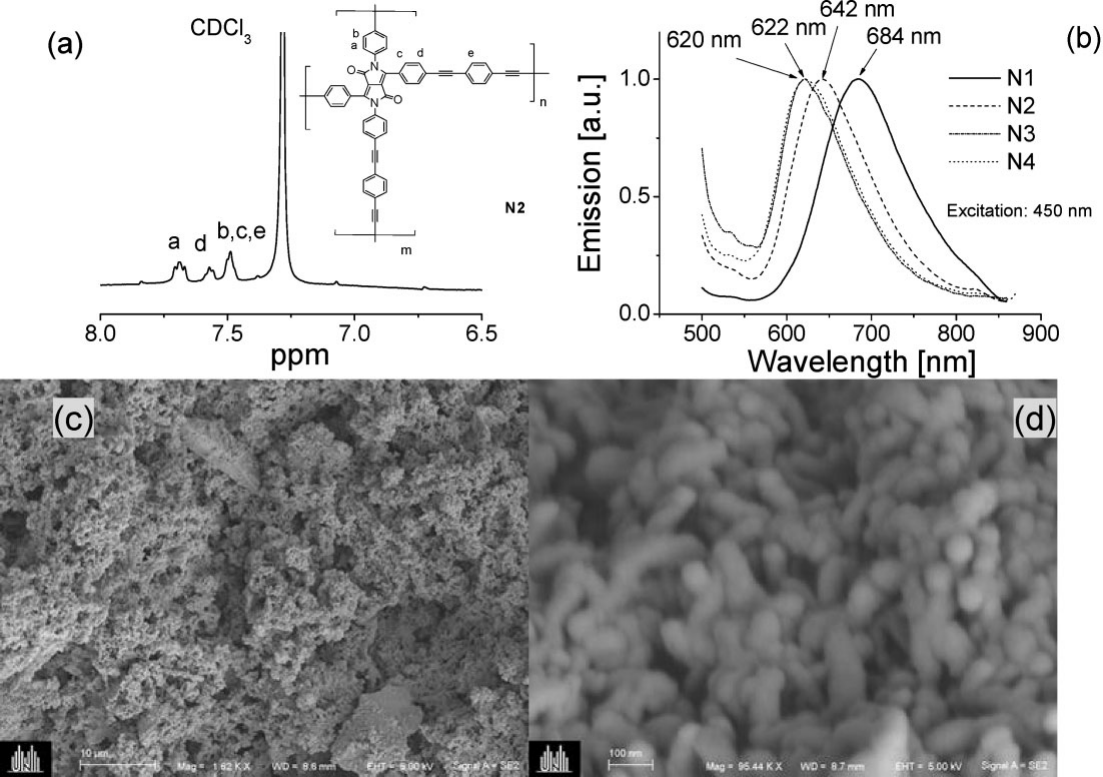
a) HR-MAS-NMR spectrum of N2. b) Fluorescence spectra of N1–4. c) SEM image with a scale bar of 10 µm and d) SEM image with a scale bar of 100 nm.

^1^H NMR spectra of cross-linked polymers swollen in deuterated chloroform were determined using the HR-MAS-NMR method. Similar to the starting compound *t-*BrDPP, the polymer networks display typical broad signals of aromatic protons of the phenyl rings attached to the DPP units. For N2 and N3 the aromatic protons of the comonomers appear in the range from 6.8 to 8.0 ppm (Figure 1a and S3).

The FT-IR spectra of the polymers N1 to N4 show stretching modes similar to that of the starting compound *t-*BrDPP. From 2 900 to 3 400 cm^−1^ C–H stretching bands appear. A typical C–O stretching mode at about 1 690 cm^−1^ and a C–C stretching mode at 1 600 cm^−1^ are observed. In addition, N2, N3, and N4 show the C≡C stretching mode at about 2 200 cm^−1^ (see Figure S5 in the Supporting Information).

The fluorescence spectra of the polymers N1–4 are displayed in Figure 1b. It shows broad fluorescence bands with maxima in the range from 620 to 684 nm. Compared to the fluorescence spectrum of the starting compound *t-*BrDPP with an absorption maximum at 524 nm, bathochromic shifts between 100 and 160 nm are observed. This indicates an extension of the π-conjugated system. Polymer N1 shows the largest bathochromic shift of 162 nm with a fluorescence maximum at 684 nm, while polymers N2–4 only show similar fluorescence maxima in the range from 620 and 642 nm. This indicates a hypsochromic effect of the comonomers on the optical properties of the polymers. N1 was obtained by a homo-coupling of the starting compound *t-*BrDPP. A better polymerization degree could be achieved, which could lead to a larger extended conjugation. Polymers N2–4 obtained by Yamamoto coupling contain ethynyl groups in the polymer main chains, the fluorescence maxima could not be as largely shifted as for N1. This is in agreement with a report on polymer networks based on a spirobifluorene building block published by Weber and Thomas.^23^

The scanning electron microscopy (SEM) images in Figure 1c and d clearly show a porous surface of the polymer network N2, and the pore size is in the nanometer range.

**Figure 2:**
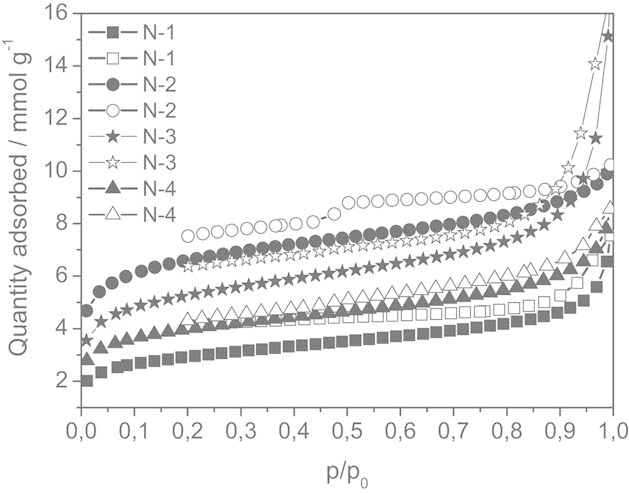
Nitrogen sorption isotherms for poly-DPP networks N1–4 (full symbols: adsorption, open symbols: desorption).

Surface areas and pore size distributions were measured for nitrogen adsorption and desorption at 77.3 K (Table 1). The nitrogen isotherms are shown in Figure 2. They reveal BET surface areas in a range from 200 to 500 m^2^ · g^−1^. According to IUPAC classifications,^24^ the adsorption isotherms suggest that the polymers are microporous structured networks of type I. Polymer N2 shows the largest BET surface area of 477 m^2^ · g^−1^ among the polymer networks, with a slight N_2_ hysteresis appearing in the desorption branch. The pore size distribution curves derived from the Barret–Joyner–Halenda (BJH) method^25^ are displayed in the Supporting Information (Figure S3). This indicates that the pores are mainly located in a mesopore range between 2 and 50 nm. The total pore volumes *V*_pore/tot_ at a relative pressure of *P/P*_0_ = 0.99 were determined to be in the range from 0.23 to 0.52 cm^3^ · g^−1^. The micropore volumes *V*_pore/micro_ were calculated in the range from 0.05 to 0.13 cm^3^ · g^−1^ using the *t*-plot method. The microporosities of the networks are in the range between 17 and 38% with network N2 showing the highest porosity (Table 1). This indicates the microporous nature of the networks. Assuming that the N_2_ gas is only adsorbed in monolayers, the surface areas were also calculated using the Langmuir method (data listed in Table 1).

## Conclusion

In summary, for the first time we have introduced fluorescent DPP into conjugated microporous networks with BET surface areas from 210 to 477 m^2^ · g^−1^ using nickel-or palladium-catalyzed cross-coupling reactions. The microwave-assisted polycondensation reactions offer a quick alternative preparation route to the conjugated polymer networks. The networks may be useful for the detection of reactive gases such as NO_*x*_, which might be able to quench the luminescence of the polymer.

## Notes

Detailed facts of importance to specialist readers are published as ”Supporting Information”. Such documents are peer-reviewed, but not copy-edited or typeset. They are made available as submitted by the authors.
