# Supplementary material for: Conjugated Microporous Networks on the Basis of 2,3,5,6-Tetraarylated Diketopyrrolo[3,4-c]pyrrole
Source: Macromol Rapid Commun. 2011 Apr 15;32(11):825–30. doi: 10.1002/marc.201100045 (PMC3810720; doi:10.1002/marc.201100045)
Supplement: Supplementary file 1 [file marc0032-0825-sd1.pdf]

Copyright WILEY-VCH Verlag GmbH & Co. KGaA, 69469 Weinheim, Germany, 2011.

Supporting Information

for *Macromol. Rapid Commun.*, 2011, 32, 825.

Conjugated Microporous Networks on the Basis 2,3,5,6-Tetraarylated Diketopyrrolo[3,4-c]pyrrole

Kai Zhang,[1] Bernd Tieke,[*1] Filipe Vilela,[2] and Peter J. Skabara [2]

[1]Department of Chemistry, University of Cologne, Luxemburger Str. 116, D-50939 Cologne, Germany

[2]WestCHEM, Department of Pure and Applied Chemistry, University of Strathclyde, Glasgow G1 1XL, United Kingdom

Introduction

In the supplement, synthetic routes of the starting compound **M1** and the networks **N1 - 4**, ^{1}H- and ^{13}C-NMR spectra of monomer ***t*-BrDPP**, HR-MAS-NMR spectra of the networks, SEM images of the networks, and pore size distributions are shown.

Experimental Section.

2,3,5,6-tetrakis(4-bromophenyl)pyrrolo[3,4-c]pyrrole-1,4(2H,5H)-dione ***t*-BrDPP** was synthesized according to literature procedures.[27] All other chemicals and solvents were purchased from commercial sources and used without further purification. Air- and/or water-sensitive reactions were conducted under nitrogen using dry solvents. Microwave assisted syntheses were carried out using a Biotage Initiator Sixty EXP Microwave System.

Physical measurements. ^{1}H and ^{13}C NMR spectra were recorded on a Bruker DPX instrument at 400 and 100 MHz; chemical shifts are given in parts per million. IR-FT spectra for the characterization of the compounds were recorded on a Perkin-Elmer FTIR spectrometer. Elemental analyses were obtained on a Perkin-Elmer 2400 elemental analyzer. Absorption spectra were measured on a Unicam UV 300 spectrophotometer. Fluorescence spectra were recorded on a Perkin-Elmer spectrometer. The porous properties of the polymer networks were investigated by nitrogen adsorption and desorption at 77.3 K using an ASAP2020 volumetric adsorption analyzer (Micrometrics Instrument Corporation). Samples were degassed at 100 °C overnight under vacuum before analysis.

Synthesis. 2,3,5,6-Tetrakis(4-bromophenyl)pyrrolo[3,4-c]pyrrole-1,4(2H,5H)-dione (M1). 3,6-Bis(4-bromophenyl)furo[3,4-c]furan-1,4-dione[27b] (500 mg, 1.12 mmol), *p*-bromo-aniline (576 mg, 3.36 mmol), dicyclohexylcarbodiimide (692 mg, 3.36 mmol) and trifluoroacetic acid (6 µl, 0.07 mmol) were dissolved in 250 ml chloroform and stirred for 3 d at room temperature. The solvent was removed, and the residue washed with methanol. After filtering, the red colored product was purified by column chromatography (toluene). The product was recrystallized from methanol as red crystals with a red solid state fluorescence. Yield: 466 mg (55 %). ^{1}H-NMR (400 MHz, $CDCl_3$): δ = 7.56 (d, aromatic, 4H), 7.51 (d, aromatic, 8H), 7.07 (d, aromatic, 4H). ^{13}C-NMR (100 MHz, $CDCl_3$): δ = 132.6, 132.0, 131.0, 129.1. UV/Vis

(dichloromethane): 338, 477, 503 nm. Fluorescence (dichloromethane): 544, 600 nm. FT-IR (cm^{-1}): 1694 (C=O), 1615 and 1485 (C=C).

Network N1. In a vial, 2,3,5,6-tetrakis(4-bromophenyl)pyrrolo[3,4-c]pyrrole-1,4(2H,5H)-dione (**M1**) (200 mg, 0.26 mmol), $Ni(COD)_2$ (343 mg, 1.25 mmol), 2,2'-dipyridyl (183 mg, 1.25 mmol) and cyclooctadiene (135 mg, 1.25 mmol) were dissolved in 10 ml dry DMF. The mixture was degassed and heated under nitrogen at 100 °C for 1 h in the microwave synthesizer. After cooling, the precipitated solid was filtered off, washed with acetone, DCM, 1 M HCl and water until acid-free. The red solid was dried under vacuum yielding 90 mg (83 %). The solid was absolutely insoluble in common organic solvents tested. HR-MAS-NMR: (500 MHz, $CDCl_3$): δ = 7.75 - 7.05 (m, aromatic H). FT-IR (cm^{-1}): 1688 (C=O), 1603 and 1492 (C=C). Fluorescence (solid): λ_{max} at 684 nm.

Network N2. In a vial, 2,3,5,6-tetrakis(4-bromophenyl)pyrrolo[3,4-c]pyrrole-1,4(2H,5H)-dione **M1** (200 mg, 0.26 mmol), 1,4-diethynylbenzene (66 mg, 0.52 mmol), tetrakis(triphenylphosphine)palladium(0) (18 mg, 0.016 mmol), copper(I) bromide (2.3 mg, 0.016 mmol) were dissolved in a mixture of DMF (5 ml) and Et_2N. The reaction mixture was degassed and heated under nitrogen at 100 °C for 1 h in the microwave synthesizer. The red solid precipitated was filtered off, washed with acetone, DCM, 1 M HCl and water until acid-free. The product was dried under vacuum yielding 128 mg (82 %). The solid was absolutely insoluble in common organic solvents tested. HR-MAS-NMR: (500 MHz, $CDCl_3$): δ = 7.65 - 7.10 (m, aromatic H). Fluorescence (solid): λ_{max} at 642 nm.

Network N-3. The procedure from **N-2** was followed except that 4,4'-diethynylbiphenyl was used instead of 1,4-diethynylbenzene, giving a red solid yielding 79 %. The solid was absolutely insoluble in common organic solvents tested. HR-MAS-NMR: (500 MHz, $CDCl_3$): δ = 7.65 - 7.10 (m, aromatic H). Fluorescence (solid): λ_{max} at 622 nm.

Network N-4. The procedure from **N-2** was followed except that 1,3,5-triethynylbenzene was used instead of 1,4-diethynylbenzene, giving a red solid yielding 80 %. The solid was absolutely insoluble in common organic solvents tested. HR-MAS-NMR: (500 MHz, $CDCl_3$): δ = 7.65 - 6.90 (m, aromatic H). Fluorescence (solid): λ_{max} at 620 nm.

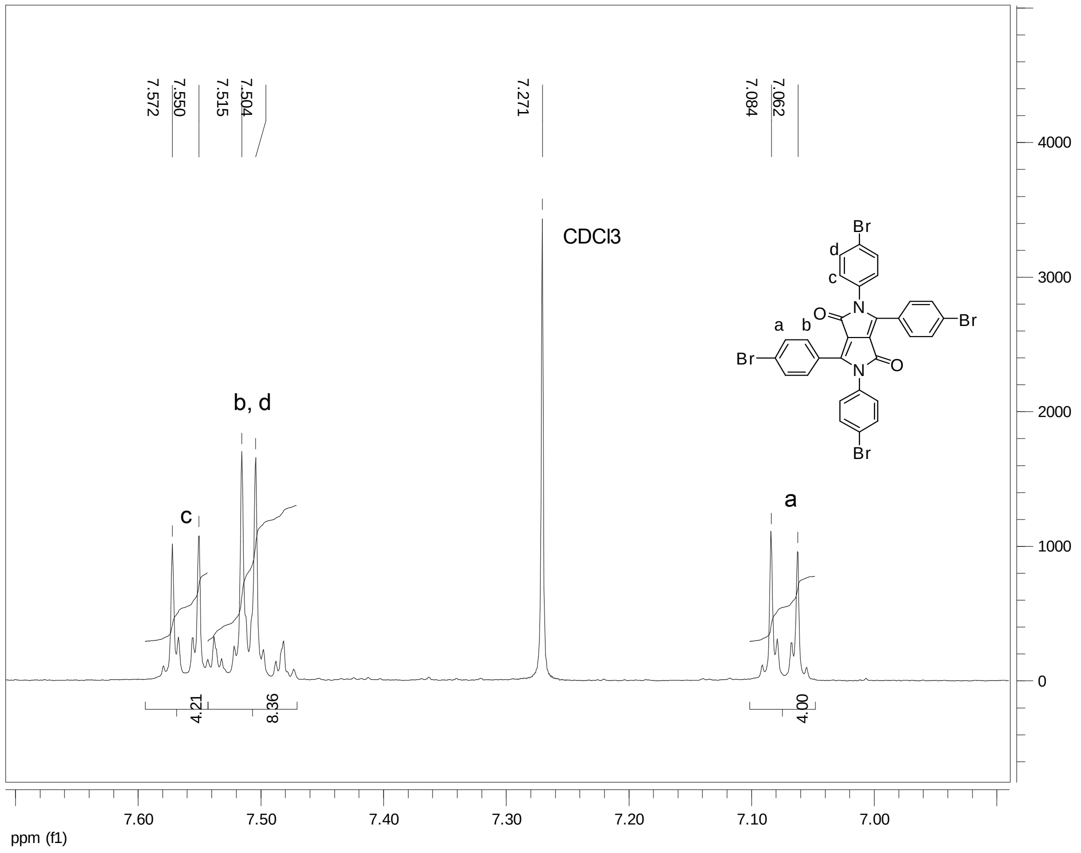

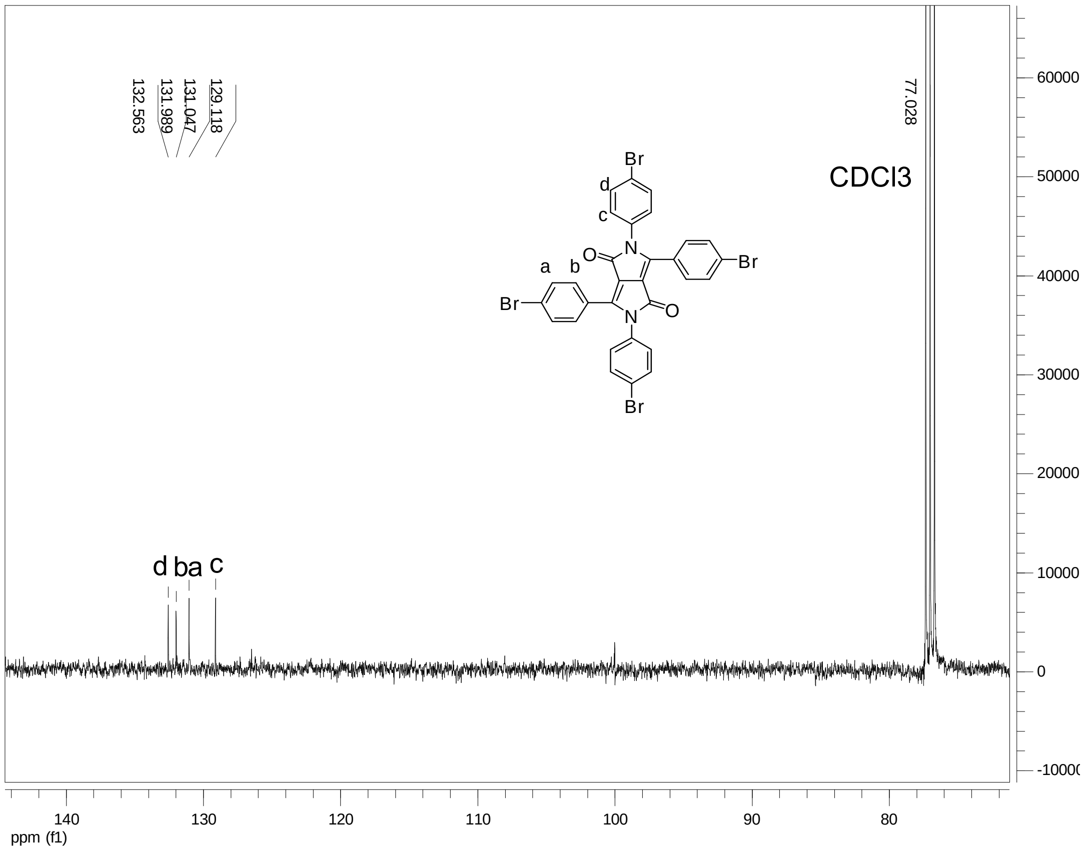

Fig. S1: ^{1}H and ^{13}C NMR spectra of monomer **M1**.

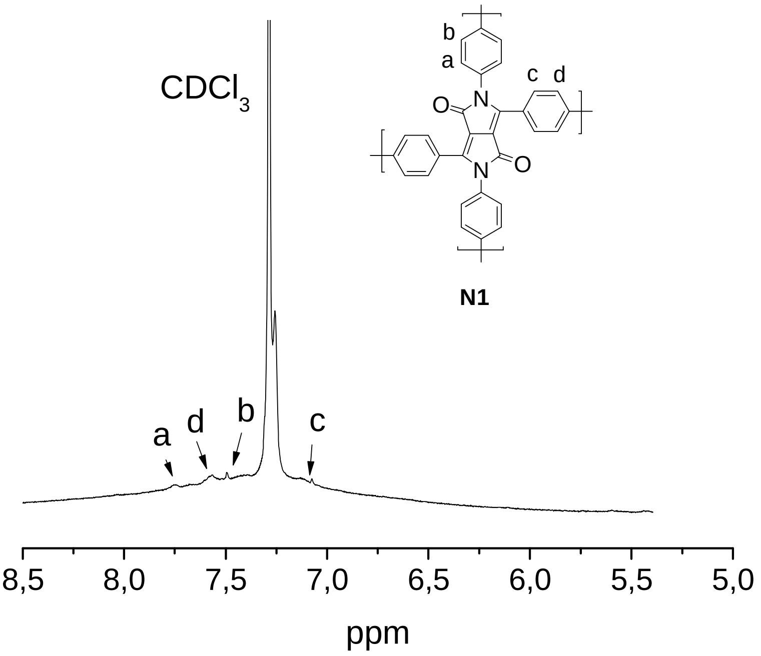

Fig. S2: HR-MAS-NMR spectrum of **N1**.

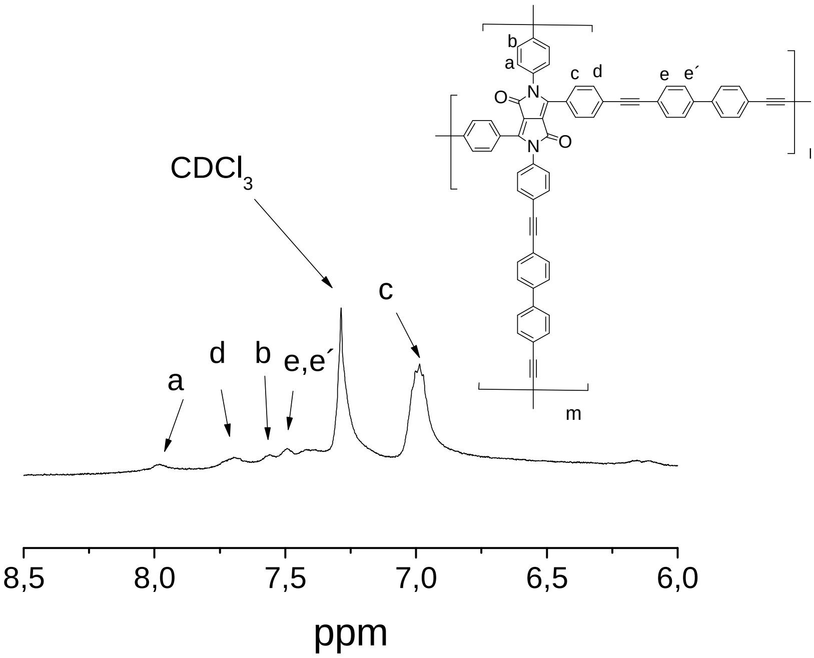

Fig. S3: HR-MAS-NMR spectrum of **N3**.

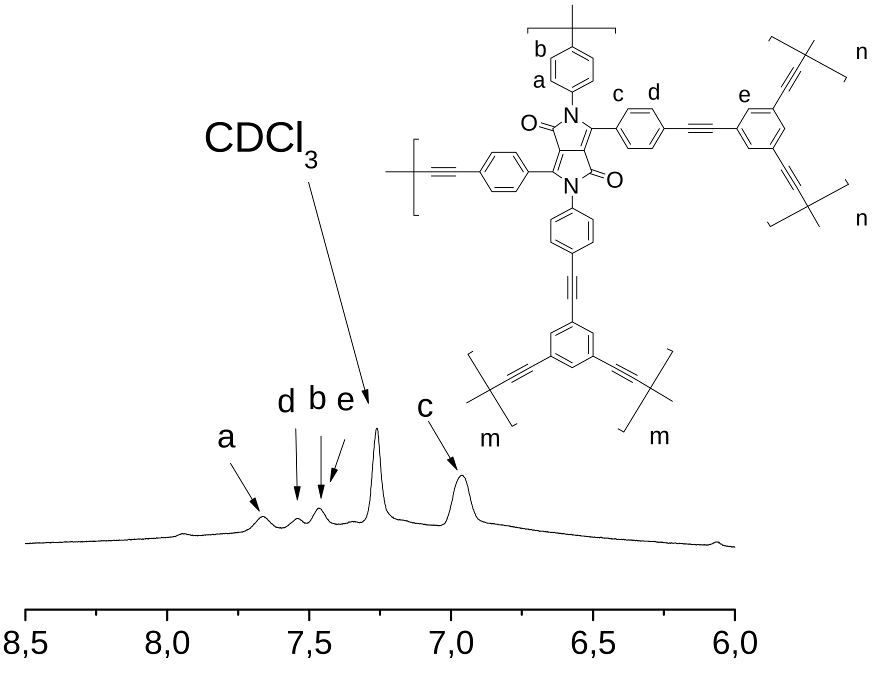

Fig. S4: HR-MAS-NMR spectrum of **N4**.

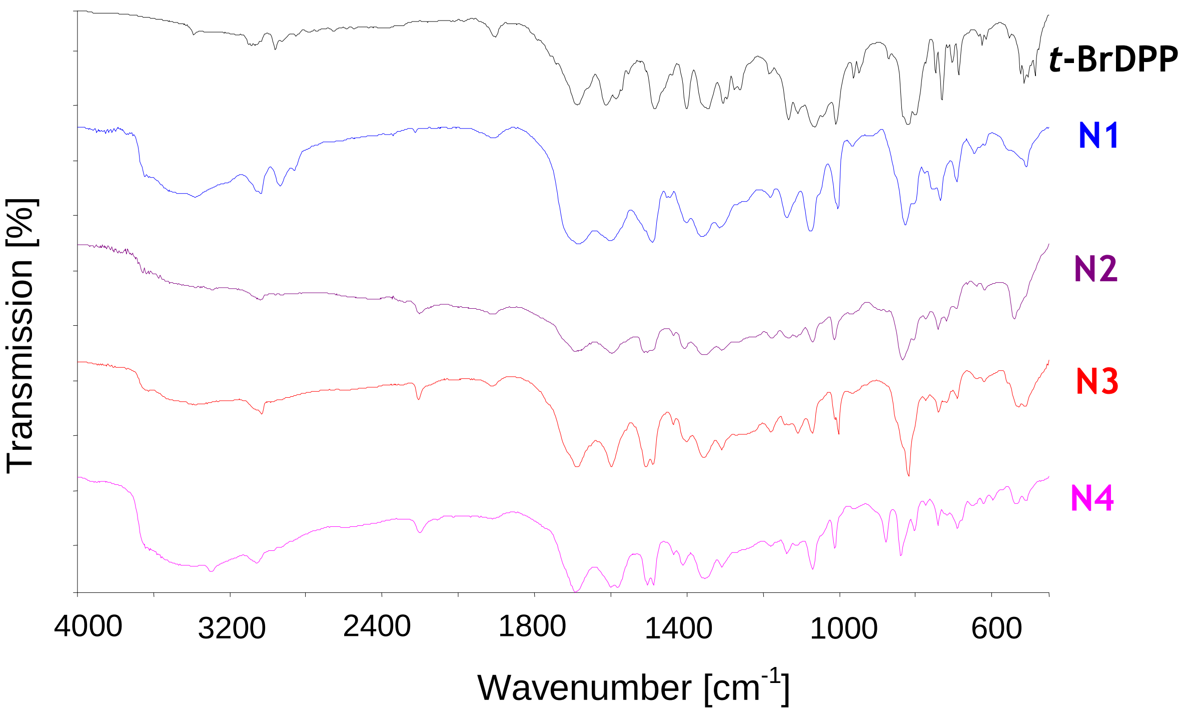

Fig. S5. FT-IR spectra of ***t*-BrDPP** and **N1-4.**

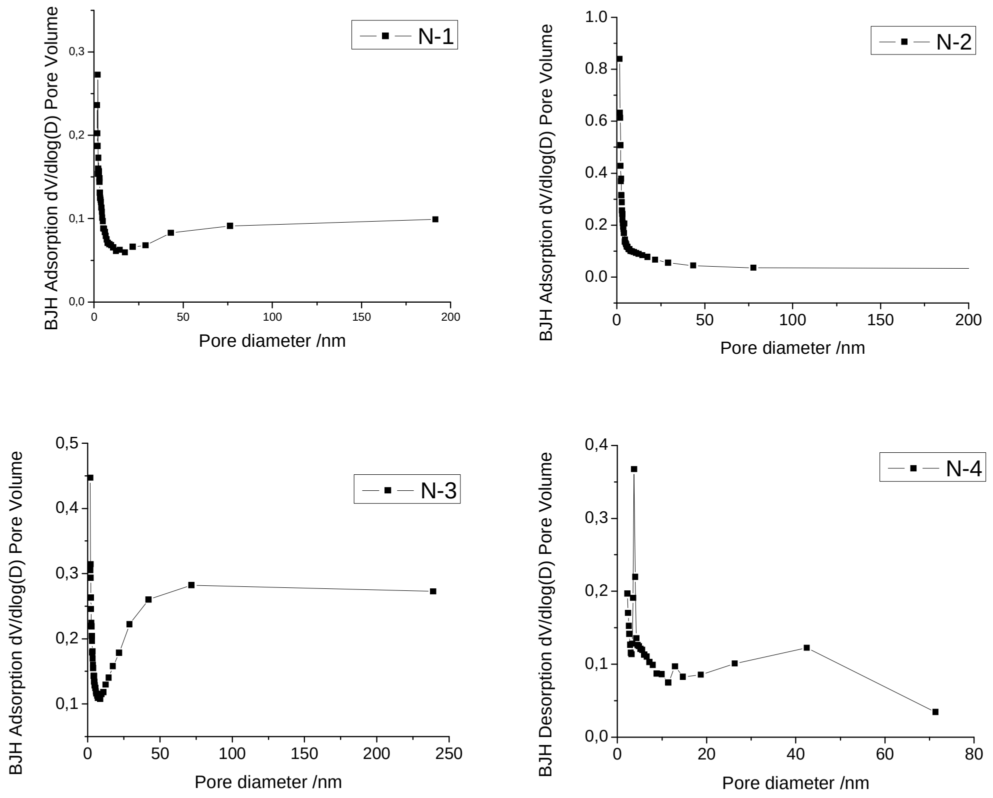

Fig. S6. Pore size distributions of network **N1 - 4.**

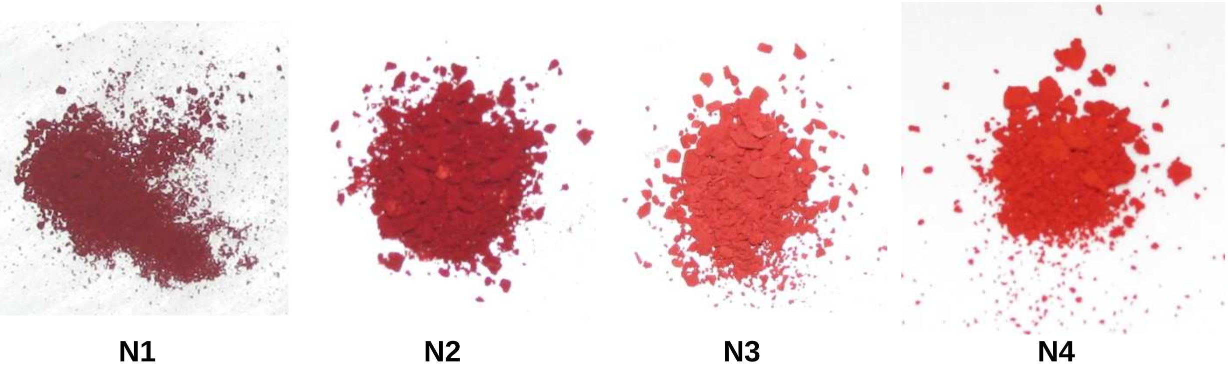

Fig. S7. Photographs of the insoluble polymer networks.

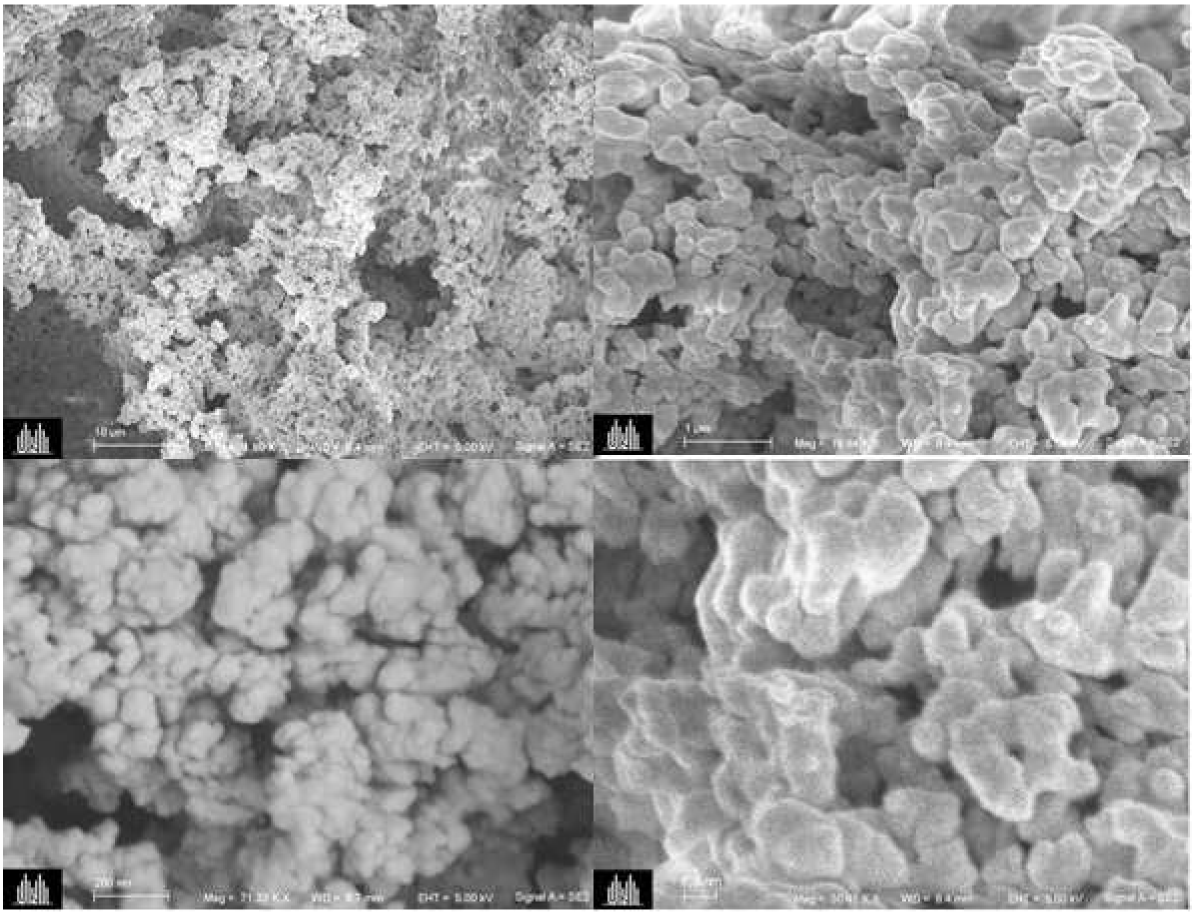

Fig. S8. SEM image of network N1, (scale: top left: 10 μm, top right: 1 μm; bottom left: 200 nm; bottom right: 100 nm).

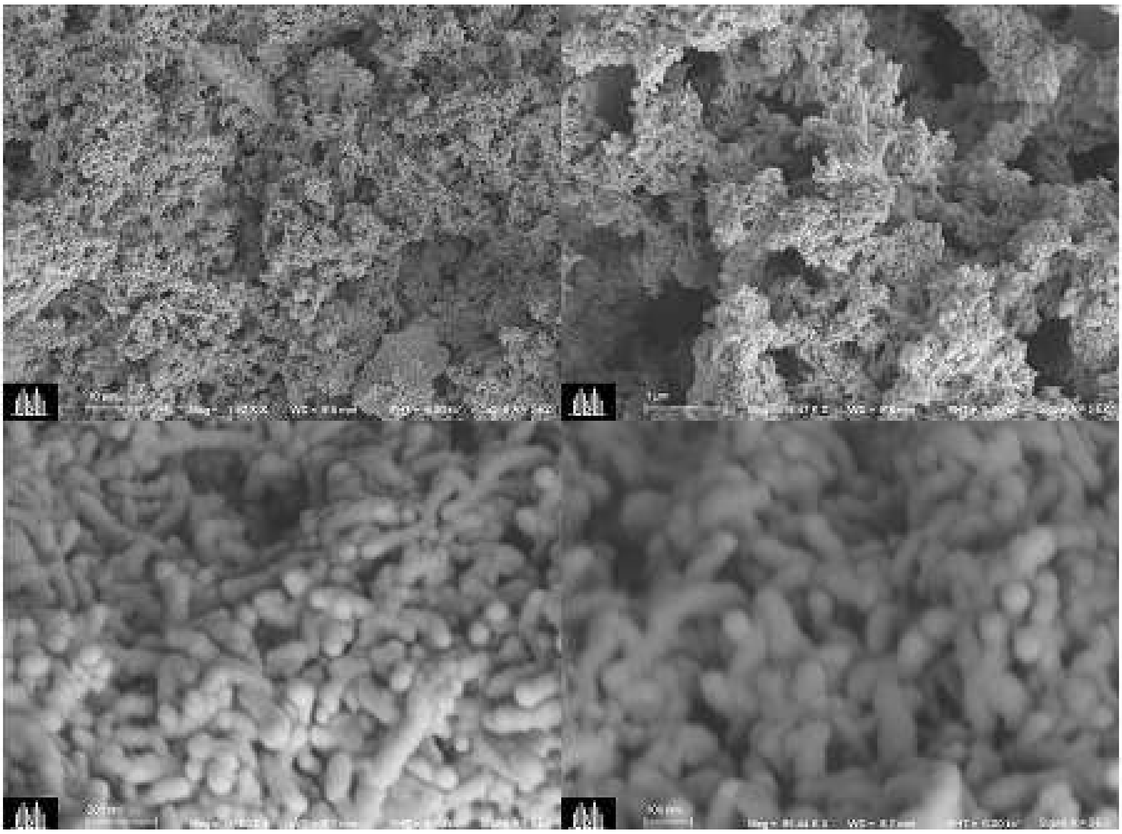

Fig. S9. SEM image of network N2, (3cale: top left: 10 μm, top right: 1 μm; bottom left: 200 nm; bottom right: 100 nm).

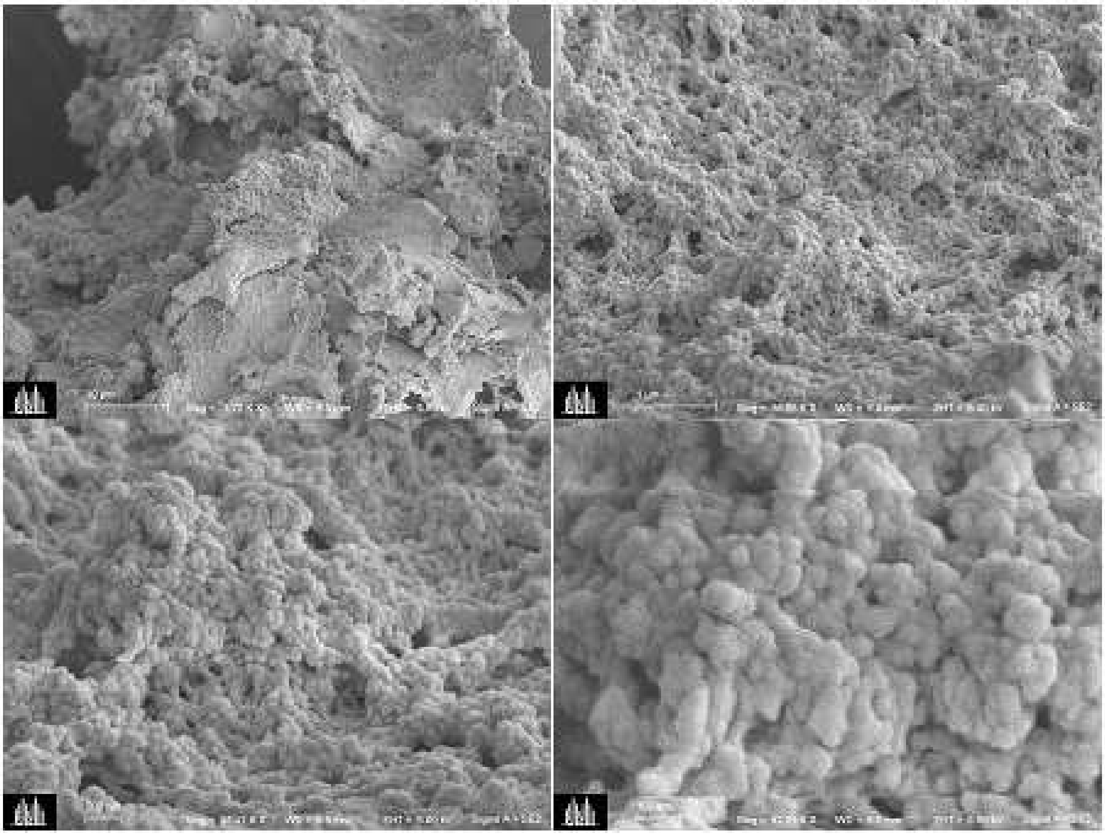

Fig. S10. SEM image of network N3, (scale: top left: 10 μm, top right: 1 μm; bottom left: 200 nm; bottom right: 100 nm).

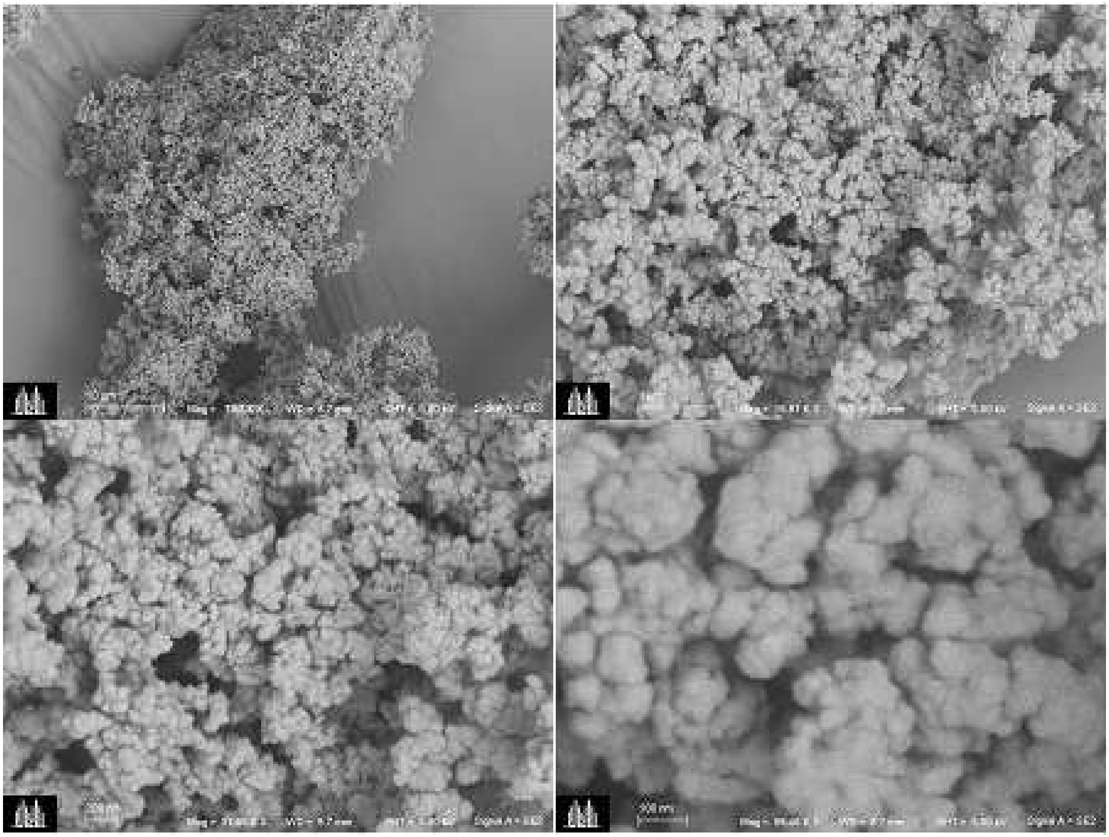

Fig. S11. SEM image of network N4, (scale: top left: 10 μm, top right: 1 μm; bottom left: 200 nm; bottom right: 100 nm).
